# In Vitro Investigation of Insulin Dynamics During 4 Hours of Simulated Cardiopulmonary Bypass

**DOI:** 10.1213/ANE.0000000000007106

**Published:** 2024-06-11

**Authors:** Thilo Schweizer, Caroline M. Nossen, Barbara Galova, Christof Schild, Markus Huber, Lia Bally, Andreas Vogt, Matthias Siepe, Michael Nagler, Kady Fischer, Dominik P. Guensch

**Affiliations:** From the * Department of Anaesthesiology and Pain Medicine, Inselspital, Bern University Hospital, University of Bern, Bern, Switzerland; † Department of Cardiac Surgery, Inselspital, Bern University Hospital, University of Bern, Bern Switzerland; ‡ University Institute of Clinical Chemistry, Inselspital, Bern University Hospital and University of Bern, Bern, Switzerland; § Department of Diabetes, Endocrinology, Nutritional Medicine and Metabolism, Inselspital, Bern University Hospital and University of Bern, Bern Switzerland.

## Abstract

**BACKGROUND:**

Hyperglycemia is common in patients undergoing cardiovascular surgery with cardiopulmonary bypass. We hypothesize that intraoperative hyperglycemia may be, at least partially, attributable to insulin loss due to adhesion on artificial surfaces and/or degradation by hemolysis. Thus, our primary aim was to investigate the loss of insulin in 2 different isolated extracorporeal circulation circuits (ECCs), that is, a conventional ECC (cECC) with a roller pump, and a mini-ECC (MiECC) system with a centrifugal pump. The secondary aim was to assess and compare the relationship between changes in insulin concentration and the degree of hemolysis in our 2 ECC models.

**METHODS:**

Six cECC and 6 MiECC systems were primed with red packed blood cells and thawed fresh-frozen plasma (1:1). Four additional experiments were performed in cECC using only thawed fresh-frozen plasma. Human insulin (Actrapid) was added, targeting a plasma insulin concentration of 400 mU/L. Insulin concentration and hemolysis index were measured at baseline and hourly thereafter. The end points were the change in insulin level after 4 hours compared to baseline and hemolysis index after 4 hours. The insulin concentration and hemolysis index were analyzed by means of a saturated linear mixed-effect regression model with a random offset for each experiment to account for the repeated measure design of the study, resulting in mean estimates and 95% confidence intervals (CIs) of the primary end points as well as of pairwise contrasts with respect to ECC type.

**RESULTS:**

Insulin concentration decreased by 63% (95% CI, 48%–77%) in the MiECC and 92% (95% CI, 77%–106%) in the cECC system that contained red blood cells. Insulin loss was significantly higher in the cECC system compared to the MiECC (*P* = .022). In the cECC with only plasma, insulin did not significantly decrease (−4%; 95% CI, −21% to 14%). Hemolysis index in MiECC increased from 68 (95% CI, 46–91) to 76 (95% CI, 54–98) after 4 hours, in cECC from 81 (95% CI, 59–103) to 121 (95% CI, 99–143). Hemolysis index and percent change of insulin showed an excellent relationship (r = −0.99, *P* < .01).

**CONCLUSIONS:**

Our data showed that insulin levels substantially decreased during 4 hours of simulated cardiopulmonary bypass only in the ECC that contained hemoglobin. The decrease was more pronounced in the cECC, which also exhibited a greater degree of hemolysis. Our results suggest that insulin degradation by hemolysis products may be a stronger contributor to insulin loss than adhesion of insulin molecules to circuit surfaces.

## Graphical abstract

**Figure:**
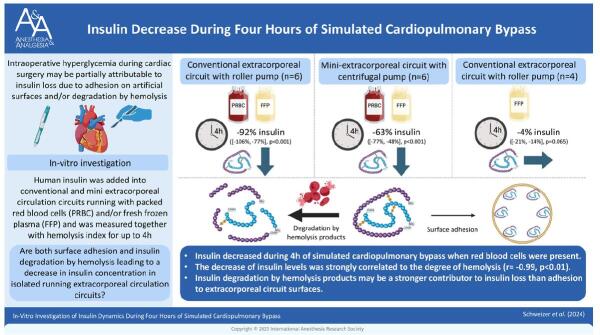

**KEY POINTS**

- **Question:** Are the changes in insulin concentrations in an extracorporeal circulation circuit (ECC) related to surface adhesion or insulin degradation?
- **Findings:** Insulin concentration decreased significantly only in ECC systems containing red blood cells, which was correlated to the degree of hemolysis in the system.
- **Meaning:** Our results favor insulin degradation by hemolysis rather than insulin adhesion to the ECC surfaces as an explanation for insulin loss during cardiopulmonary bypass.

Approximately 80% of all patients undergoing cardiovascular surgery on cardiopulmonary bypass (CPB) suffer from perioperative hyperglycemia >7.8 mmol/L (140 mg/dL), and even 60% of nondiabetic patients develop perioperative stress hyperglycemia.^1^ This is a problem, as intraoperative hyperglycemia is an independent risk factor for morbidity and mortality in both diabetic and nondiabetic patients.^2^ Hyperglycemia is associated with an increased probability of postoperative adverse events, such as postoperative infection rates and with an increased risk of neurocognitive dysfunction in nondiabetic patients.^3–5^ In patients undergoing coronary artery bypass graft (CABG) surgery on CPB, insulin requirements were 30% higher than in patients that underwent off-pump CABG.^6^ This turns the spotlight on the CPB as an important factor for poor glycemic control.

Response to surgical stress and its related release of cytokines, catecholamines and glucocorticoids, but also preexisting insulin resistance or diabetes are known causes of increased insulin resistance and therefore hyperglycemia. Hypothermia and nonpulsatile flow during CPB may impair insulin production; however, studies showed that C-Peptide levels as a measurement of endogenous insulin production were not markedly diminished in patients on CPB.^7–10^ Heparin and the repetitive administration of glucose with the cardioplegic solution, which is used to stop the heart, are further increasing glucose levels.^9,11^

Robust studies about insulin dynamics during CPB are lacking, and CPB-related depletion of insulin as a contributor to hyperglycemia has not been studied in detail before. Two main mechanisms are potential culprits for an insulin decrease during CPB. One proposed mechanism is, that insulin molecules may bind to CPB surfaces such as the tubing, the oxygenator and the reservoir, thereby becoming biologically unavailable.^12,13^ Urban et al^14^ showed that relevant adhesion of insulin molecules to CPB-tubing takes place; however, the currently used bio-coating is known to reduce the adhesion up to 48% compared to uncoated tubing. The second proposed mechanism for elevated insulin degradation is CPB-related hemolysis, as hemolysis products have been proven to cause degradation of insulin in vitro.^15–18^ On CPB, red blood cells are prone to hemolysis, promoted by shear stress that occurs through suctioning blood from the surgical field, but also through the pumps of the CPB circuit.^19^

The aim of this study was to investigate insulin decrease in an in vitro CPB-simulation experiment using 2 different isolated extracorporeal circulation circuit (ECC) systems. Our hypothesis was, that both surface adhesion and insulin degradation may lead to a decrease in insulin concentration in isolated running ECC systems.

## METHODS

### Study Design and End Points

No sample size analysis was conducted as the study is exploratory in nature. This study used packed red blood cells (PRBC) and fresh-frozen plasma (FFP) from anonymized donors to prime the circuits. These units were officially removed from use in patient care by the transfusion laboratory of the University Hospital Bern due to reasons like interruption in the cooling chain or exceeding the expiry date. Informed consent of the donors could not be obtained as the origin of these units were not traceable for us. The Ethics Committee of the Canton Bern issued a waiver for this study and deemed the study ethically acceptable.

The primary end point was the change in insulin concentration after 4 hours of simulated CPB in conventional ECC (cECC) and mini-ECC (MiECC) systems. Secondary end points were the quantification of hemolysis, the association between the change in insulin concentration and hemolysis, and the effect of the ECC type on these measurements.

### Experiments in a Simulated CPB Model

#### Preparations

**Figure 1.**
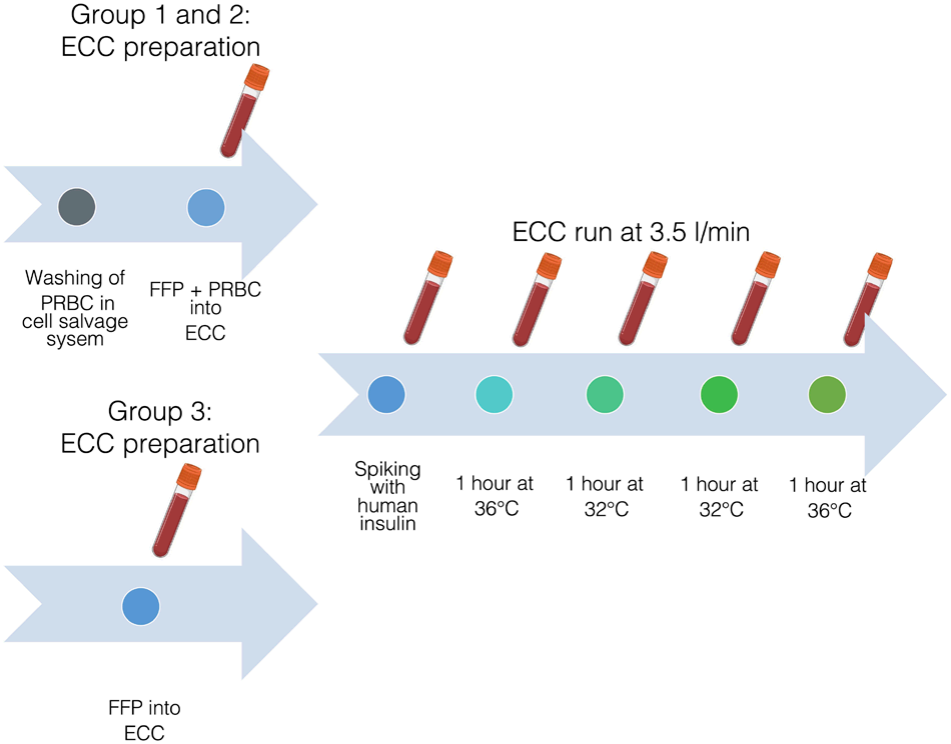
Visualization of the preparation and the ECC runs. ECC indicates extracorporeal circulation circuit; FFP, fresh-frozen plasma; PRBC, packed red blood cells.

Sixteen experiments were conducted. As the PRBC were predominantly of older age, they were washed in a cell salvage system (CSS, autoLog Autotransfusion System, Medtronic) before they were added together with compatible FFP into the reservoir of an ECC to washout potentially biasing preexisting hemolytic and metabolic products (Figure 1). Six experiments used a cECC with a roller pump (group 1, Heart Lung Machine HL20, Getinge), and in 6 experiments we used a MiECC with a centrifugal pump (group 2, Bio-Console 560 Extracorporeal Blood Pumping Console, Medtronic/Revolution Centrifugal Blood Pump with Phisio phosphorylcholine biopassive coating, LivaNova). The cECC were primed with 2 units of PRBC and 2 units of FFP, the MiECC were primed with only 1 unit of each due to its more compact setup. In 4 additional experiments, the cECC, as the standard circuit in our institution, was primed with only 4 units of FFP, to exclude the effect of potential hemolysis (group 3). To minimize possible bias, we used the identical tubing (Custom Tubing Pack with a tube diameter of 3/8 inch and Cortiva heparin bioactive coating, Medtronic), reservoir (Inspire HVR Dual Hard Shell Venous Reservoir, Phisio phosphorylcholine biopassive coating, LivaNova) and oxygenator (Affinity Fusion Membranoxygenator with Cortiva heparin bioactive coating, Medtronic) in the cECC and MiECC, respectively. These coatings are used in our clinical systems and are intended to minimize the interaction of blood with the artificial surfaces and mimic vascular endothelium. The purpose of this coating is to reduce coagulation and inflammatory responses, but also to reduce adhesion of proteins, such as insulin, to the circuit surfaces.^14,20–22^

After filling the ECC systems with the blood products we added 5000 U of heparin (Liquemin, Drossapharm AG) to prevent clotting of the blood. An initial blood sample was acquired to measure the hemoglobin concentration, to confirm a physiologic pH and to verify the absence of insulin in the blood products.

#### In Vitro Cardiopulmonary Bypass Runs

The venous and arterial tubing of the ECCs were directly connected. Human insulin (Actrapid, Novo Nordisk A/S) was administered into the system to target a plasma insulin concentration of 400 mU/L. The ECCs were set to a flow of 3.5 L/min for the entire experiment. In our institution, a common ECC-time during cardiovascular surgery is approximately 4 hours; however, this strongly depends on the type of surgery. This way we mimicked a typical ECC-run at our site by setting the temperature first at normothermia, (36 °C), followed by 2 hours of mild hypothermia (32 °C), and finally concluding the experiment with normothermia. Insulin concentrations were measured together with hemolysis index (HI) shortly after the start of the run (baseline), and from then on hourly along with the pH. If necessary, unphysiological pH-values were corrected by administration of sodium bicarbonate. Serum insulin concentration was analyzed using a human-insulin-specific electrochemiluminescence immunoassay (Elecsys Insulin, Cobas 8000, Roche Diagnostics). To ensure accuracy and precision of the insulin measurements an insulin recovery test was performed, which was deemed acceptable (Supplemental Digital Content 1, http://links.lww.com/AA/E910). HI was measured photometrically. A HI of 100 (arbitrary units) approximately corresponds to 1 g/L (100 mg/dL) of free hemoglobin in the serum.^23^

### Data Analysis

The primary outcome (insulin levels) and HI were analyzed by means of 2 saturated linear mixed-effect regression models with a random offset for each experiment to account for the repeated measure design of the study. Inferred means and 95% confidence intervals (CI) of the outcomes at each time point are based on the regression models. Pairwise contrasts and associated 95% CI of the change in insulin levels and the HI at 4 hours among the 3 experimental groups were adjusted by Tukey’s honestly significant difference method and are presented in Supplemental Digital Content 1, http://links.lww.com/AA/E910. A Pearson’s correlation tested the linear relationship between insulin decrease and hemolysis.

**Figure 2.**
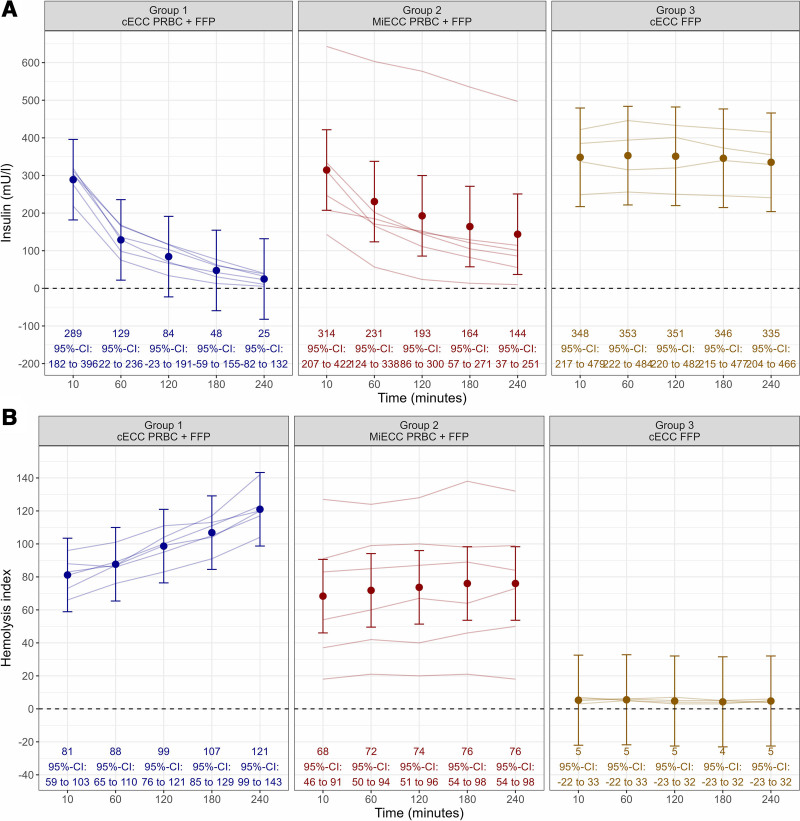
Dynamics of insulin concentration and hemolysis index in 2 different ECC systems. A, A decrease in insulin concentration was found in both ECC systems containing PRBC (groups 1 and 2), while no significant decrease in insulin concentration was detected in the experiments containing only FFP (group 3). In the cECC (group 1) a more pronounced decrease than in the MiECC (group 2) was detected. B, Hemolysis index increased over time in both ECC systems (groups 1 and 2); however, the increase was higher in the cECC (group 1). Mean and associated 95% confidence intervals based on a saturated linear mixed-effect model are shown (see “Methods” section). ECC indicates extracorporeal circulation circuit; cECC, conventional ECC; FFP, fresh-frozen plasma; MiECC, mini-ECC; PRBC, packed red blood cells.

If applicable, nonparametric tests for descriptive statistical analysis were applied. Statistical significance was defined with a 2-sided *P*-value of <.05. GraphPad Prism version 10 (GraphPad Software), IBM SPSS Statistics 26 (IBM) and R version 4.0.2 were used for statistical analysis.^24^

## RESULTS

### Experiments in a Simulated CPB Model

In group 1 (cECC with FFP/PRBC) the insulin concentration dropped from 289 (95% CI, 182–396) mU/L 10 minutes after start to 25 (95% CI, 82–132) mU/L after 4 hours. In group 2 (MiECC with FFP/PRBC), insulin decreased from 314 (95% CI, 207–422) mU/L to 144 (95% CI, 37–251) mU/L after 4 hours (Figure 2A). This corresponds to an insulin loss of 92% (95% CI, 77–106, *P* < .001) in group 1 and 63% (95% CI, 48–77, *P* < .001) in group 2, respectively (Supplemental Digital Content 1, Supplementary Figure SM1, http://links.lww.com/AA/E910). Insulin loss was significantly higher in group 1 compared with group 2 by −29% (95% CI, −54% to −4%, *P* = .022). In group 3 (cECC with only FFP), insulin did not decrease significantly (Figure 2A; 10 minutes: 348 (95% CI, 217–479) mU/L, 4 hours: 335 (95% CI, 204–466) mU/L; change of −4% (95% CI, −21% to 14%), *P* = .65).

**Figure 3.**
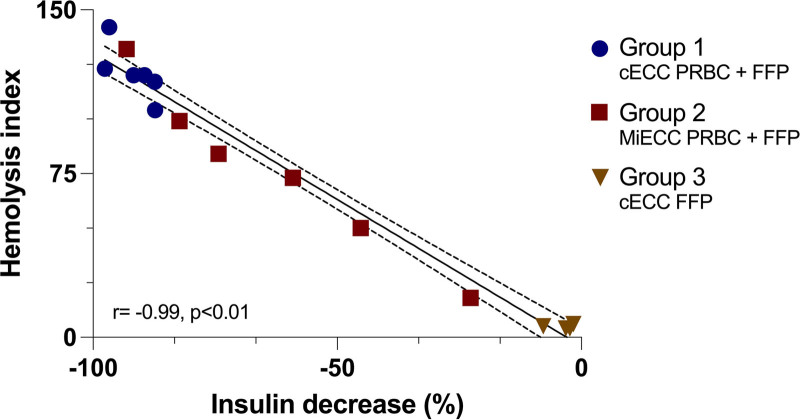
Relationship between hemolysis index and percent decrease in insulin after 4 h of simulated cardiopulmonary bypass. An excellent correlation between decrease of insulin concentration and degree of hemolysis was found (r = –0.99, *P* < .01). cECC indicates conventional extracorporeal circulation circuit; FFP, fresh-frozen plasma; MiECC, mini-ECC; PRBC, packed red blood cells.

In group 1 HI increased from 81 (95% CI, 59–103) to 121 (95% CI, 99–143) after 4 hours (change: 40 [95% CI, 35–45], *P* < .001; Supplemental Digital Content 1, Supplementary Figure SM2, http://links.lww.com/AA/E910). HI in group 2 increased from 68 (95% CI, 46–91) to 76 (95% CI, 54–98) after 4 hours (change: 8 [95% CI, 3–13], *P* = .005). The change of HI over time was higher in group 1, compared with group 2 (32 [95% CI, 23–41], *P* < .001; Supplemental Digital Content 1, Supplementary Figure SM2, http://links.lww.com/AA/E910). HI and percent change of insulin showed a very high correlation (Figure 3; r = −0.99, *P* < .01).

## DISCUSSION

Our data showed that insulin levels significantly decreased during 4 hours of simulated CPB independent of patient factors in ECC systems that contained red blood cells. That decrease was more profound in the cECC, which also exhibited a greater degree of hemolysis. The cECC with only FFP showed no decrease in insulin levels, which favors the hypothesis of insulin degradation over that of adhesion.

### Insulin Adhesion to ECC Surfaces

Earlier investigations suggested that adhesion of insulin molecules to surfaces takes place in a relevant amount in infusion bottles, intravenous infusion sets, and surfaces of CPB machines.^12,14,25^ However, a remarkably difference to our study was, that these studies used normal saline as a solvent of the insulin and therefore, the insulin was the only polypeptide that was capable of binding to surfaces. The latter studies reported an increasing saturation of the binding sites over time. The FFP used in our study contains relevant amounts of other polypeptides that can competitively block binding sides of ECC tubing surfaces and limit the binding of insulin to these surfaces. This might explain, why we did not see significant insulin decrease in the FFP-only experiments and therefore relevant insulin adhesion to surfaces. Of note, Urban et al^14^ reported that bio-coating can reduce insulin adhesion on polyvinyl choride plastic tubing up to 48%. Our tubing and the oxygenator were bio-coated, together with the polypeptides in the FFP that may have limited insulin adhesion sufficiently. This assumption is supported by the results from the experiments containing only FFP, which showed no significant loss of insulin after 4 hours of simulated CPB.

### Insulin Degradation

Multiple studies showed, that hemolysis products can cause decline of insulin concentration in vitro.^15–18^ However, the exact mechanisms are still controversially discussed. Studies showed, that the insulin-degrading enzyme insulysin, which is released from red blood cells during hemolysis, is breaking down insulin molecules.^16,17^ One study suggested, that degradation of insulin is likely caused by the globin part of the hemoglobin.^15^ In this study the authors used pure hemoglobin in the same concentration of other hemolytic blood samples in their experiments and found a degradation of insulin in the absence of insulysin. Subsequent experiments suggested that the thiol group of the globin caused a splitting of the disulfide bonds of insulin. As we did not quantify insulysin activity nor insulin fragments, it remains elusive to what extend the decrease in insulin concentration in our model was caused by insulysin or degradation by the globin chain.

### Limitations

The roller pump is an important cause of hemolysis during cardiac surgery with CPB, the centrifugal pump is known to produce hemolysis to a smaller extend.^19^ However, pericardial suctioning is a major source of hemolysis.^26^ A limitation of this study is, that we did not include a simulation of pericardial suctioning. Therefore, during surgery, hemolysis might even be higher than in our in vitro study. In addition, this study did also not account for patient factors, such as a potential modulation of endogenous insulin production as well as mechanisms of insulin clearance that determine the normal insulin-half-live in vitro.

### Clinical Impact

The aspect, that hemolysis induced degradation of insulin might play a part in glycemic control and might contribute to the need of higher insulin doses during cardiac surgery on CPB, is new. Clinicians should be aware of the finding, that a higher degree of hemolysis can correspond with a decrease in insulin levels. Not only the pumps can cause hemolysis, but also is pericardial suctioning a major contributor for hemolysis due to the high shear stress. Tighter bleeding control and using of CSS may mitigate this effect. The need for higher insulin doses should be expected when using a cECC compared to a MiECC system due to the increased hemolysis. Newer cECC systems nowadays also use centrifugal pumps, which may reduce the problem of hemolysis. It is now warranted to investigate insulin dynamics along with hemolysis in patients undergoing cardiac surgery with CPB to also account for intrinsic patient factors and factors associated with cardioplegia, suctioning, CSS as well as pharmacologic management from anesthesia providers. These findings should then be incorporated into pharmacokinetic models to guide clinicians on appropriate insulin dosing and improve glycemic control and subsequently patient outcome.

## CONCLUSIONS

A significant decrease in insulin concentration was detected in a simulated CPB model over 4 hours only, when red blood cells were present. Insulin decrease was higher in a cECC than in an MiECC, which correlated highly with the degree of hemolysis. These findings suggest that insulin degradation by hemolysis products contributes much stronger to insulin decrease than adhesion of insulin molecules to circuit surfaces in current ECC systems. It is now warranted to investigate insulin dynamics along with hemolysis in patients undergoing cardiac surgery on CPB in an observational study.

## DISCLOSURES

**This manuscript was handled by:** Karsten Bartels, MD, PhD, MBA.

## Supplementary Material


