# Supplementary material for: In Vitro Investigation of Insulin Dynamics During 4 Hours of Simulated Cardiopulmonary Bypass
Source: Anesth Analg. 2024 Jun 11;141(2):267–72. doi: 10.1213/ANE.0000000000007106 (PMC12220572; doi:10.1213/ANE.0000000000007106)
Supplement: Supplementary file 1 [file ane-141-267-s001.docx]

**In-Vitro Investigation of Insulin Dynamics During Four Hours of Simulated Cardiopulmonary Bypass: Supplemental Material**

Thilo Schweizer, MD^1^, Caroline M. Nossen, B Med^1^, Barbara Galova, EBCP^2^, Christof Schild, PhD^3^, Markus Huber, PhD^1^, Lia Bally, MD, PhD^4^, Andreas Vogt, MD^1^, Matthias Siepe, MD^2^, Michael Nagler, MD, PhD^3^, Kady Fischer, PhD^1^, Dominik P. Guensch, MD^1*^

^1^ Department of Anaesthesiology and Pain Medicine, Inselspital, Bern University Hospital, University of Bern, Bern, Switzerland
^2^ Department of Cardiac Surgery, Inselspital, Bern University Hospital, University of Bern, Bern Switzerland
^3^ University Institute of Clinical Chemistry, Inselspital, Bern University Hospital and University of Bern, Bern, Switzerland
^4^ Department of Diabetes, Endocrinology, Nutritional Medicine and Metabolism, Inselspital, Bern University Hospital and University of Bern, Bern Switzerland

**Declaration of interests:** The authors declare they have no competing interests.

**Funding:** Institutional funding is provided by Bern University Hospital, Department of Clinical Chemistry and the Department of Anaesthesiology and Pain Medicine Scientific Fund.

**Running title:** Insulin loss in simulated cardiopulmonary bypass

***Corresponding Author:**

Prof. Dominik Guensch, MD, DESA, FEACVI,

Department of Anaesthesiology and Pain Medicine, Inselspital, Bern University Hospital, University of Bern, Freiburgstrasse 10, 3010 Bern, Switzerland, Tel: +41-(0)31-632 0377, Fax: +41 (0)31-632 0554, E-Mail: dominik.guensch@gmail.com

Twitter: @DGuensch

**Keywords:** Cardiopulmonary Bypass; Insulin; Cardiovascular Surgery; Hyperglycemia; Extracorporeal circulation; Heart Lung Machine; Glycemic control

**In-vitro insulin recovery experiment**

Methods:

An in-vitro recovery experiment for insulin involves assessing the accuracy and precision of insulin measurement techniques in-vitro and is normally expressed as percent-recovery by the assay of known added amount of analyte in the sample. In this study it was used to evaluate the performance of insulin assay and technique used for insulin quantification. The recovery of human recombinant insulin (Actrapid®, Novo Nordisk, Bagsværd, Denmark) was tested according to Westgard JO and Quam EF in FFP (Octaplas®, Octapharma, Lachen, Switzerland) and a 50%/50% mix of FFP and PRBC in the Institute of Clinical Chemistry in our institution.^1^ For this purpose, Insulin Actrapid® was prediluted to 40000 mU/L in saline. Then, the matrices were spiked with insulin to yield insulin concentrations of 0, 20, 100 and 200 mU/L. Insulin concentrations were then assessed with the same immunoassay as in the main experiments.

Results:

The insulin concentration was below the limit of detection (<0.4 mU/L) in native FPP and in the 50%/50% FFP/PRBC mix. For the targeted insulin concentrations of 0, 20, 100 and 200 mU/L in native FFP, the recovery was <0.4, 14.2, 90.3 and 179 mU/L, corresponding to a recovery of 71, 90 and 90% for the samples with added insulin. This resulted in a mean recovery of 84%. In the 50%/50% FFP/PRBC mix recovery was <0.4, 18.5, 91.2 and 172mU/L, what corresponds to a recovery of 93, 91 and 86%, which averages to a mean recovery of 90%.

Discussion:

The acceptable recovery range is often defined as 70-120%.^2^ Thus, the recovery of 84% in FFP and 90% in the FFP/PRBC mix were deemed acceptable. This means that 84% and 90% of the insulin added to the samples could be detected with the test. Therefore, the recovery experiments confirmed the validity of this test.


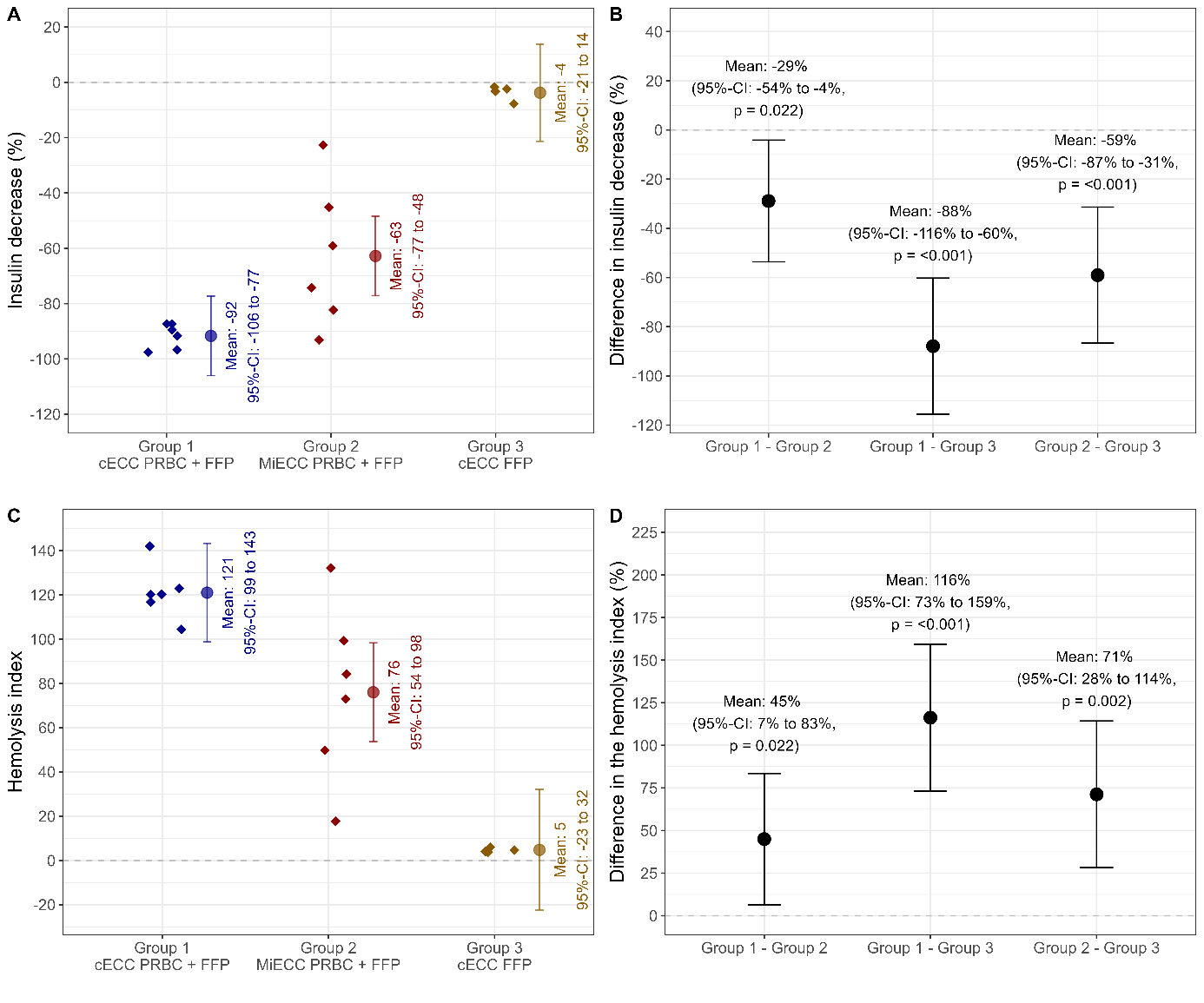


**Supplementary Figure SM1: Insulin and Hemolysis Index after 4h of Simulated Cardiopulmonary Bypass**

Mean and 95% confidence intervals are shown for all panels. **A:** Insulin concentration decreased significantly in circuits where red blood cells were present (group 1 and 2), but not for group 3 where only fresh frozen plasma (FFP) was present. **B:** Pairwise comparisons of the insulin decrease after 4h between the groups demonstrates that there was a significant difference in insulin loss between all three groups, with the highest loss observed in the conventional extracorporeal circulation circuit (cECC) with packed red blood cells (PRBC) and FPP, in comparison to both the mini-extracorporeal circulation circuit (MiECC) with the same contents and cECC with FPP only. The bottom row depicts similar analysis for the measured hemolysis index with relevant hemolysis in groups 1 and 2 where red blood cells were present (**C**). **D:** Pairwise comparisons of the hemolysis index after 4h demonstrates significantly different hemolysis between all three groups.


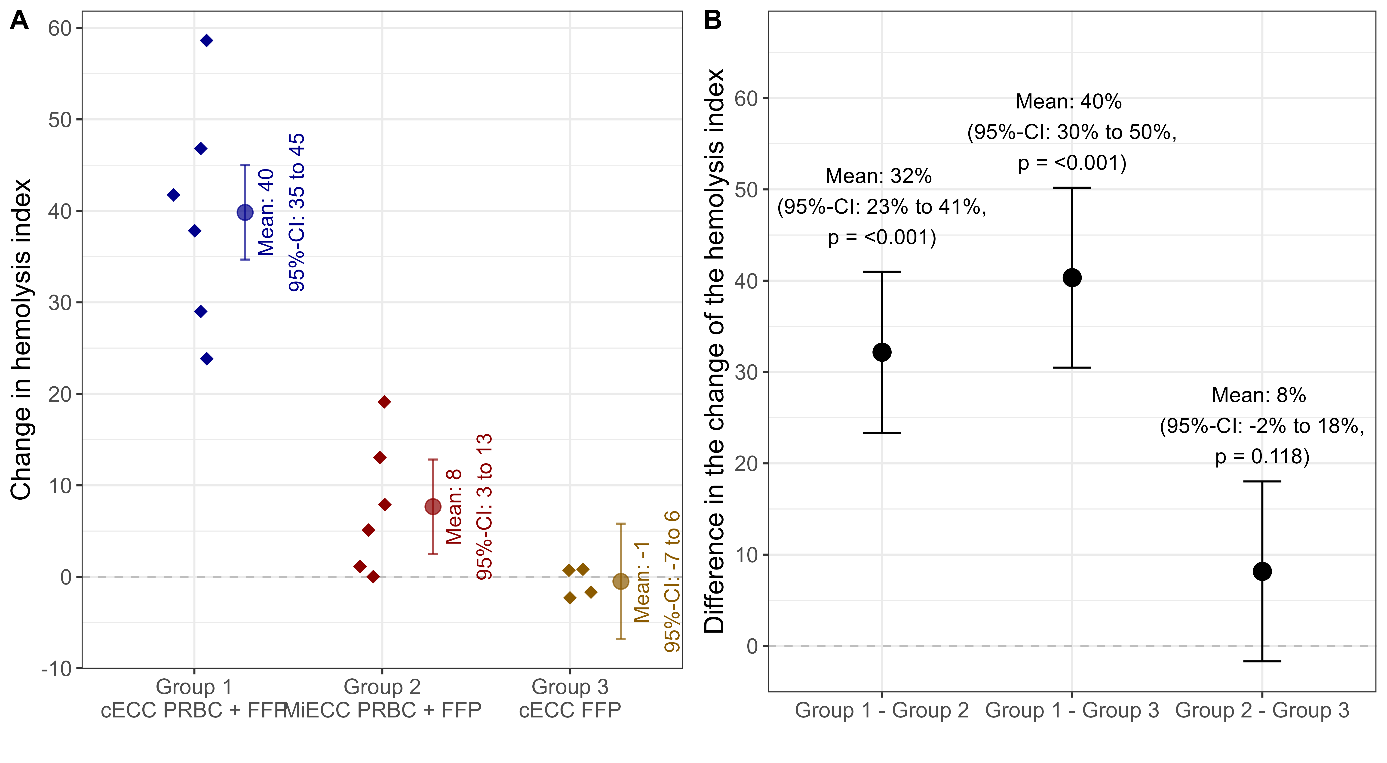


**Supplementary Figure SM2. Change in hemolysis index over 4h of simulated cardiopulmonary bypass**.

Mean and 95% confidence intervals are displayed. **A:** The difference in the hemolysis index between baseline and the 4h timepoint were calculated to support the findings in SM1. This analysis accounted for any baseline variations in hemolysis. **B:** Pairwise comparisons of the change of the hemolysis index over 4h between the groups are shown. Conventional extracorporeal circulation circuit (cECC), fresh frozen plasma (FFP), mini-extracorporeal circulation circuit (MiECC), packed red blood cells (PRBC).

References:

1. Westgard JO, Barry PL, Quam EF, Ehrmeyer SS. *Basic Method Validation: Training in Analytical Quality Management for Healthcare Laboratories*. Westgard Quality Corporation; 1999. https://books.google.ch/books?id=8LwhAQAAMAAJ

2. Abraham J. International Conference On Harmonisation Of Technical Requirements For Registration Of Pharmaceuticals For Human Use. In: Tietje C, Brouder A, eds. *Handbook of Transnational Economic Governance Regimes*. Brill | Nijhoff; 2010:1041-1053. doi:10.1163/ej.9789004163300.i-1081.897
